# State of abdominal CT datasets: A critical review of bias, clinical relevance, and real-world applicability

**DOI:** 10.1371/journal.pdig.0001567

**Published:** 2026-08-10

**Authors:** Saeide Danaei, Zahra Dehghanian, Elahe Meftah, Nariman Naderi, Seyed Amir Ahmad Safavi-Naini, Faeze Khorasanizade, Hamid R. Rabiee

**Affiliations:** 1 Department of Computer Engineering, Data Science and Machine Learning Lab (DML), Sharif University of Technology, Tehran, Iran; 2 Data-Driven and Digital Health (D3M), The Charles Bronfman Institute for Personalized Medicine, Icahn School of Medicine at Mount Sinai, New York, New York, United States of America; 3 Research Institute for Gastroenterology and Liver Diseases, Shahid Beheshti University of Medical Sciences, Tehran, Iran; 4 Advanced Diagnostic and Interventional Radiology Research Center (ADIR), Tehran University of Medical Science, Tehran, Iran; Instituto Politécnico Nacional Escuela Superior de Medicina: Instituto Politecnico Nacional Escuela Superior de Medicina, MEXICO

## Abstract

This review systematically searched publicly available abdominal CT datasets and critically evaluates suitability for artificial intelligence (AI) applications in clinical settings. We examined 45 publicly available abdominal CT datasets (47,049 studies). Across all 45 datasets, we found substantial redundancy (51% case reuse) and a Western/geographic skew (75.3% from North America and Europe). A bias assessment was performed on the 22 datasets with more than 100 cases; within this subset, the most prevalent high-risk categories were racial bias (with a score of 16 out of 22) and selection bias (with a score of 15 out of 22), both of which may undermine model generalizability across diverse healthcare environments—particularly in resource-limited settings. To address these challenges, we propose targeted strategies for dataset improvement, including multi-institutional collaboration, adoption of standardized protocols, and deliberate inclusion of diverse patient populations and imaging technologies. These efforts are crucial in supporting the development of more equitable and clinically robust AI models for abdominal imaging.

## Author summary

Abdominal CT scans are widely used by doctors to detect diseases and guide treatment decisions, but analyzing these images is challenging and time-consuming. Artificial intelligence (AI) has the potential to make this process faster and more reliable. However, AI systems are only as good as the datasets they are trained on. In this study, we reviewed 45 publicly available abdominal CT datasets, covering 47,049 scans, to understand how well they support real-world medical applications. We found that many datasets share the same cases, most of which come from North America and Europe, and important groups of patients and diseases are underrepresented. These imbalances mean that AI tools built from current datasets may not work equally well for all patients, especially in countries with different healthcare settings. We suggest strategies to make future datasets more useful and fair, including collaboration across hospitals worldwide, better diversity in patient data, and more consistent standards for imaging and labeling. By improving datasets, researchers and clinicians can create AI systems that are more accurate, equitable, and beneficial for patients everywhere.

## 1 Introduction

Abdominal computed tomography (CT) imaging is central to the diagnosis, treatment monitoring, and surgical planning of conditions affecting the liver, pancreas, spleen, kidneys, and other abdominal organs [1]. Accurate interpretation of these images demands specialized radiological expertise that remains scarce across many healthcare systems, contributing to diagnostic delays and suboptimal outcomes, particularly for rare or complex pathologies [2]. Artificial intelligence (AI) offers a promising avenue to bridge this gap, with models demonstrating potential for automated organ segmentation, early disease detection, and quantitative biomarker extraction in both emergency and resource-constrained settings [3,4]. Organ segmentation, the precise delineation of anatomical boundaries in CT volumes, underpins many of these downstream applications by enabling reproducible volumetric assessment and tumor burden quantification [5]. Yet the clinical reliability of such models is fundamentally bounded by the quality, diversity, and representativeness of the datasets on which they are trained and validated. Models developed on biased or narrowly characterized datasets frequently fail to generalize across patient populations, imaging protocols, and clinical environments, limiting both their safety and their adoption in routine practice [6,7].

Bias in abdominal CT datasets is not a single deficiency but a chain of vulnerabilities that accumulates across the dataset lifecycle [8]. The chain often begins at the point of patient recruitment, where single-center collection or narrow inclusion criteria produce cohorts that overrepresent specific demographics, disease stages, or referral patterns, while excluding the clinical heterogeneity that AI models will inevitably encounter in deployment [9]. The problem deepens at the acquisition stage, where differences in scanner hardware, multi-phase contrast protocols, and reconstruction parameters, choices that directly govern tissue contrast and spatial resolution in abdominal imaging, introduce systematic technical variation that models may learn as spurious features rather than genuine anatomy. Even when recruitment and acquisition are well controlled, annotation practices introduce a further layer of fragility. Inter-reader variability in organ boundary delineation, inconsistent lesion labeling conventions, and reliance on semi-automated segmentation without rigorous human validation can embed subjective disagreement into what models treat as ground truth [10]. The cumulative effect is that a dataset may appear large and well-structured yet carry compounding distortions that silently propagate through every model trained on it.

Despite growing recognition of these challenges, the bias literature in medical imaging has predominantly examined downstream model behavior, identifying performance disparities after training has already occurred [9,11]. Landmark studies have revealed racial [12], gender [13], and geographic [14] biases in clinical algorithms, while subsequent work has documented systematic underdiagnosis in underserved populations across chest radiography and dermatology [15,16]. Yet these investigations consistently evaluate bias at the model output, after training has already absorbed whatever distortions the data contain. The datasets themselves, the upstream resources that determine what biases models can inherit, have received far less scrutiny. In particular, no review has jointly assessed the demographic composition, annotation practices, organ and pathology coverage, and reuse patterns of publicly available abdominal CT datasets, despite the fact that these shared resources underpin a substantial and growing fraction of segmentation and detection model development. Without such an audit, the field risks building successive generations of models on foundations whose limitations remain uncharacterized.

This critical review evaluates the current landscape of publicly available abdominal CT datasets with emphasis on dataset composition, annotation quality, demographic representation, and multi-dimensional bias assessment. We systematically characterize dataset reuse patterns and quantify bias across eight predefined categories to identify concrete limitations and actionable opportunities for improvement. By doing so, we aim to inform the development of more representative, clinically relevant, and methodologically transparent datasets that can support equitable and trustworthy AI systems for abdominal imaging.

## 2 Materials and methods

This study was designed as a comprehensive critical review of publicly available abdominal CT datasets, with the aim of characterising their suitability for developing, validating, and deploying AI models in clinical practice. The information extraction and evaluation was structured around four dimensions that collectively determine a dataset’s fitness for purpose: composition and redundancy, annotation practices and quality, demographic and geographic representation, and potential bias. These dimensions were selected because each independently constrains the generalisability of models trained on a given resource, and their combined profile determines the conditions under which a dataset can be reliably applied [9,17,18]. The following subsections describe the search strategy, inclusion criteria, data extraction procedures, and bias assessment framework in turn.

### 2.1 Dataset identification

We conducted a systematic search to identify publicly available abdominal CT datasets across multiple databases and repositories. The primary sources were Google Scholar, PubMed, Scopus, and institutional repositories including the National Institutes of Health imaging collections and The Cancer Imaging Archive (TCIA). These sources were selected for their complementary coverage of the medical imaging research literature and established dataset hosting platforms. The initial search was conducted between between October and December 2024 using keyword combinations that included “abdominal CT dataset,” “abdominal organ segmentation,” “CT imaging public dataset,” and “abdominal CT annotation” and individual abdominal organ names combined with “segmentation” or “annotation” (e.g., liver, kidney, spleen, and pancreas). Two subsequent update searches were performed between December 2025 and January 2026 to capture newly released datasets and incorporate revised keyword combinations informed by findings from the preceding round. Because this work evaluates datasets as resources rather than synthesising intervention effects or diagnostic accuracy estimates, formal protocol registration was not pursued. The complete extraction sheet, including all search parameters and inclusion decisions, is publicly available at the repository linked in the Data availability statement.

Beyond the database-driven search, we performed a cross-reference analysis by examining the data source descriptions and reference lists of all initially retrieved datasets to trace constituent datasets that had been reused, merged, or re-annotated within composite collections such as AbdomenCT-1K [19], FLARE23 [20], and AbdomenAtlas 1.1 [21]. We also screened dataset hosting platforms outside the conventional academic literature, including Kaggle, Zenodo, and Grand Challenge, to capture publicly released resources not indexed through standard bibliographic channels. Datasets identified through these supplementary routes were subjected to the same inclusion criteria described below.

### 2.2 Study selection and inclusion criteria

We screened candidate datasets against five predetermined criteria designed to ensure clinical relevance and technical suitability for AI model development. First, datasets were required to contain CT images in volumetric (three-dimensional) format, thereby excluding two-dimensional extracted slices and bounding-box-only resources as a fundamentally different class of imaging data with distinct use cases. Second, at least one form of labelled region, such as organ contours, tumour boundaries, or anatomical landmarks, had to be provided as polygonal segmentation masks or voxel-based annotations corresponding to the full volumetric data. Third, these annotations were required to support clinically relevant tasks, including organ segmentation, anomaly detection, or disease diagnosis with clear applications in diagnostic radiology or treatment planning. Fourth, annotations had to pertain to key abdominal structures including but not limited to the liver, kidneys, spleen, pancreas, stomach, and associated vasculature. Fifth, each dataset was required to be referenced in at least one peer-reviewed publication demonstrating its use in medical imaging research or machine learning model development. This final criterion means that datasets hosted on platforms such as Zenodo or Kaggle without an accompanying formal publication were not captured, and the review therefore represents the published abdominal CT dataset landscape rather than the complete universe of available resources.

The initial search and scan identified 48 candidate datasets. After applying the inclusion criteria, we excluded three. AbdomenAtlas 1.0 was superseded by version 1.1 [21], which contains substantially more public cases (9,262 versus 5,195) and enhanced annotations; only the most recent version was retained to avoid version duplication. SAROS [22], while including 900 public abdominal CT scans, annotates general anatomical regions such as subcutaneous tissue, muscle, and body cavities rather than organ-specific structures, and therefore does not meet the organ-level annotation requirement. DeepLesion [23] was excluded because it provides extracted two-dimensional image slices in PNG format with bounding-box annotations rather than 3D CT volumes with segmentation masks. (Similarly, ULS23 [24] provides cropped regions around lesions across diverse anatomical locations rather than whole-abdomen volumetric scans with organ-level annotations. While both datasets represent valuable resources for lesion detection, their format falls outside the volumetric scope of this review. A systematic evaluation of two-dimensional slice-based and bounding-box datasets constitutes a distinct effort with different quality considerations and clinical use, which we reserve for future work.).

### 2.3 Data extraction

Two independent reviewers (S.D. and E.M.) extracted data from all eligible datasets using a standardised form. In cases of disagreement, a third reviewer (SAASN) adjudicated to reach consensus. Each dataset was evaluated across six parameter domains. Dataset composition captured the total number of CT studies, the proportion of publicly available versus private cases, and temporal growth patterns. Clinical context recorded disease status, clinical indications, and disease severity distributions. Data provenance documented contributing institutions, geographic origin, and whether collection was prospective or retrospective. Imaging characteristics encompassed scanner specifications, acquisition protocols, contrast enhancement status, slice thickness, and spatial resolution. Annotation details included the organs annotated, segmentation methodology, validation procedures, and the number of annotators involved. Demographic information covered age distribution, sex ratio, racial and ethnic composition, and reported comorbidities.

### 2.4 Bias assessment framework

To evaluate dataset suitability for developing generalisable AI models, we performed a structured bias assessment on all datasets containing 100 or more publicly available cases. Smaller datasets were excluded from this assessment because their limited disease coverage and case spectrum produce high-risk ratings across nearly all categories by default, diminishing the discriminative value of categorical risk classification [17].

#### 2.4.1 Bias categories and operational definitions.

No established quality checklist exists for evaluating publicly available medical imaging datasets as training resources for AI. We therefore developed a bias assessment framework by adapting instruments designed for related purposes, specifically PROBAST-AI for prediction model risk-of-bias assessment [18], the quality criteria for medical imaging AI datasets proposed by Kocak et al. [9], and the TRIPOD+AI reporting guidelines for AI-based diagnostic and prognostic studies [17]. From these sources, we derived seven bias categories, each rated on a three-tier scale (low, moderate, or high risk), with categories for which the primary documentation provided insufficient information recorded as “not available" (NA). Complete operational definitions, decision rules, and classification examples are provided in S1 Table. The complete dataset identification, screening, and inclusion process is detailed in S1 Fig. The seven categories and their rating criteria are summarised below.

**Spectrum bias (case representation)** evaluates the severity range and clinical variation within the positive class. Datasets were rated from low risk (most clinically important variations covered) to high risk (heavily skewed representation with many significant presentations missing).

**Selection bias (non-case representation)** evaluates the spectrum of variation and differential diagnoses within the non-case group. Datasets were rated from low risk (most relevant differentials included) to high risk (many differentials absent or only normal cases as comparators).

**Racial bias** evaluates the coverage of racial and ethnic groups relative to the intended scope of deployment. Datasets were rated from low risk (most major groups represented) to high risk (single racial group or single-institution origin in a racially homogeneous catchment area).

**Demographic bias** evaluates the balance of sex and age distributions relative to the clinical population. Datasets were rated from low risk (approximately balanced distributions) to high risk (heavily skewed sex distribution or an entire clinically relevant age group absent).

**Technical bias** evaluates the diversity of imaging hardware, acquisition protocols, and contrast administration relative to routine clinical practice. Datasets were rated from low risk (multiple manufacturers with varied protocols) to high risk (single scanner model or exclusively high-specification research equipment).

**Labelling bias** evaluates the rigour and consistency of the annotation pipeline. Datasets were rated from low risk (at least two human reviewers with complete expert validation) to high risk (minimal expert supervision or fully automated pipelines without reported accuracy against expert-validated data).

**Temporal bias** evaluates the time span and period of data collection. Datasets were rated from low risk (collection spanning more than five years, capturing natural temporal variation) to high risk (collection within a single year or entirely within the COVID-19 pandemic period of 2019–2021).

Taken together, these seven categories capture three higher-order threats to clinical applicability. Case representation risk (spectrum, labelling, and selection bias) concerns whether a model trained on a given dataset will reliably distinguish pathological from nonpathological findings across the case mix encountered in routine practice. Population bias (racial and demographic bias) concerns systematic performance degradation within specific demographic subgroups that are underrepresented or absent during training. Domain shift risk (technical, labelling, and temporal bias) concerns generalisation failure when a model is deployed across institutions, imaging infrastructure, or time periods that differ from those in the training set. This grouping guided our interpretation of the bias landscape and is revisited in the Discussion.

#### 2.4.2 Bias evaluation and quality assessment workflow.

Three evaluators (S.D., N.N., and E.M.) independently assessed all datasets meeting the 100-case threshold. S.D. is a researcher with a Master’s degree in medical imaging, N.N. is a physician with research experience in medical image analysis, and E.M. is a general practitioner with research experience in medical imaging. Prior to independent assessment, a joint calibration session was held to ensure consistent interpretation of the operational definitions and rating criteria.

For each dataset, each evaluator assigned a risk rating (low, moderate, or high) for all seven bias categories based solely on primary documentation sources, including published data descriptors, constituent dataset papers for composite collections, and repository documentation when formal descriptors were unavailable. For ratings of moderate or high risk, evaluators recorded a written rationale justifying their classification. Dataset creators were not contacted for additional information, ensuring that all assessments reflect publicly accessible documentation available to the broader research community.

Conflict was defined as disagreement between two or more of the three evaluators on a given category. Conflicting ratings were resolved by a fourth reviewer (S.A.A.S-N., a physician-researcher with four years of experience in CT and MRI segmentation) in consultation with a board-certified radiologist attending (F.K.). This resolution process ensured integration of clinical, technical, and radiological perspectives while maintaining classification consistency across datasets with varying documentation quality.

Inter-rater reliability was quantified using Krippendorff’s α (ordinal distance weighting) as the primary metric and mean pairwise quadratic-weighted Cohen’s κ as a complement. For IRR computation, categories rated as NA were treated as Moderate risk, while the derived overall classification was excluded from agreement calculations. The overall Krippendorff’s α was 0.727 (95% CI 0.62 to 0.84), indicating substantial agreement. Per-category α values ranged from 0.199 for temporal bias to 0.820 for selection bias. Full per-category agreement statistics, pairwise rater comparisons, and annotator-versus-adjudicated-ground-truth agreement are provided in S3 Table.

#### 2.4.3 Overall bias classification.

Overall bias classifications were derived from per-category ratings using a weighted composite scoring system informed by three established principles: the multi-domain assessment structure of ROBINS-I [25], the cumulative downgrading logic of the GRADE framework [26], and the principle from QUADAS-2 that undocumented domains cannot be assumed low risk [27]. Categories recorded as “not available” (NA) were treated as Moderate risk for the purposes of score computation. For each dataset, a continuous bias score was computed as:


$$\begin{array}{c} \text{Bias Score}=\sum (\text{High-risk ratings})\times 1.0\;+\;\sum (\text{Moderate-risk ratings}+\text{NA categories})\times \frac{1}{3} \end{array}$$

Classification thresholds were anchored proportionally to the seven-category framework, such that each threshold corresponds to the fraction of assessed domains effectively compromised. Datasets scoring 3.0 or above were classified as Critical Bias, those from 2.0 to below 3.0 as High Bias, those from 1.0 to below 2.0 as Moderate Bias, and those below 1.0 as Low Bias. The full derivation and justification of the scoring formula, including worked examples and the mapping of individual categories to higher-order bias threats, are provided in S1 Table. Complete per-dataset classifications and justifications are provided in S2 Table.

## 3 Results

### 3.1 Overview of datasets

Based on the structured evaluation framework outlined in the Methodology, this section presents key findings regarding the composition, annotation practices, dataset bias, and demographic diversity of publicly available abdominal CT datasets. We analyzed 45 datasets encompassing a total of 47,049 CT studies to assess their suitability for AI-driven medical applications. Tables 1 and 2 indicate summarized details such as the number of volumes, the proportion of cases reused, pathology status, contributing centers, source countries, annotated organs, the availability of anomaly labels, and annotation methods.

**Table 1:** Dataset volume and subject information.

Dataset name	Cases (public + private)	Reused Cases	Subjects Status
SLIVER (2007) [28]	20 + 10	0	Most cases had tumors, metastasis, and cysts of different sizes in liver
3D-IRCADb (2010) [29]	22 + 0	0	Liver tumors, FNH cases
VISCERAL (2015) [30]	40 + 27	0	Bone marrow neoplasms
BTCV (2015) [31]	30 + 20	0	Cancer, post-op hernia
Colorectal-Liver-Metastases (2017) [32]	394 + 0	0	CRC with liver metastases
DenseVNet (2018) [33]	90 + 0	100% [31,34]	Healthy, liver metastases
LiTS (2018) [35]	131 + 70	10% [29]	Liver cancer, pre/post-therapy
MSD-CT – Spleen task (2018) [36]	41 + 20	0	Liver metastases post-chemo
pancreatic Cancer Survival Prediction (2018) [37]	159 + 53	0	Candidates for pancreatic cancer resection
MSD-CT – Colon task (2018) [36]	126 + 64	0	Candidates for colorectal cancer resection
SegThor (2019) [38]	40 + 20	0	NSCLC, curative radiotherapy
CHAOS (2019) [39]	20 + 20	0	Healthy donors, atypical livers
CT-ORG (2020) [40]	119 + 21	94% [35]	Liver lesions (benign/malignant) with cancers of other organs
MSD-CT – Pancreas task (2020) [36]	281 + 139	0	Candidates for pancreatic mass resection
MSD-CT – Liver task (2020) [36]	131 + 70	100% [35]	Liver cancer, pre/post-therapy
MSD-CT – HepaticVessel task (2020) [36]	303 + 140	0	Primary, metastatic liver tumors
Pancreas-CT (2016) [34]	80 + 0	0	Healthy donors, non-pancreatic cases
AbdomenCT-1K (2021) [19]	1112 + 0	95%(multiple datasets)	Various abdominal cancers
WORC – GIST dataset (2021) [41]	246 + 0	0	GIST, intra-abdominal tumors resembling GIST
WORC – CRLM dataset (2021) [41]	77 + 0	0	CRC liver metastases
Pediatric (2022) [42]	359 + 0	0	Pediatric CT cases
WORD (2022) [43]	170 + 0	12% [35]	Cancer, pre-radiotherapy
AMOS (2022) [44]	500 + 0	0	Abdominal tumors, other nonmalignant abdominal pathologies
KiPA22 (2022) [45]	100 + 30	0	Renal tumors affecting only one kidney
StageII-Colorectal-CT (2022) [46]	230 + 0	0	Stage II CRC, pre-op CTs
HCC-TACE-Seg (2022) [47]	211 + 0	0	HCC, TACE treatment cases
AutoPET (FDG-PET/CT) (2022) [48]	1014 + 150	0	Oncological cases (mostly NSCLC, lymphoma, melanoma)
DAP Atlas (2023) [49]	533 + 0	100% [48]	Cancer, tumors, and enlarged anatomical structures
Abdominal Trauma Det (2024) [50]	3551 + 723	0	Traumatic injuries
TotalSegmentator (2023) [51]	1204 + 0	0	Many organ pathology variations and also normal cases
AbdomenAtlas 1.1 (2024) [21]	9262 + 11223	60%(multiple datasets)	Normal and cancerous organs (colorectal, pancreatic)
FLARE23 (2023) [20]	4250 + 400	100% (multiple datasets)	Normal and cancerous cases
KiTS (2019) [52]	489 + 110	0	Kidney tumor or cysts suspicious of malignancy
CPTAC-PDA-Tumor-Annotations (2023) [53]	97 + 0	0	Pancreatic ductal adenocarcinoma
CPTAC-CCRCC-Tumor-Annotations (2023) [54]	55 + 0	0	Clear Cell Renal Cell Carcinoma, pre/post-treatment
CARE (2023) [55]	399 + 0	0	Rectal cancer and its surrounding normal tissue
Low-dose (2023) [56]	75 + 0	0	Liver metastasis
SEG.A. (2023)	56 + 0	100% [52]	Aortic pathologies
CT Lymph Nodes (2023) [57]	86 + 0	0	Noncancerous lymphadenopathy
Adrenal-ACC-Ki67-Seg (2023) [58]	65 + 0	0	Adrenocortical carcinoma with assessed Ki-67 index
AIMI Annotations Initiative (2024) [59]	1,231 + 0	100% [32,47,54]	Kidney and liver tumor
CURVAS (2024) [60]	20 + 70	0	Cysts and other pathologies (benign and malignant)
PANORAMA (2025) [61]	2,238 + 486	10% [34,36]	PDAC, non-PDAC: healthy pancreas, benign pancreatic lesions
WAW-TACE (2024) [62]	233 + 0	0	Treatment-naive patients with unresectable HCC treated with TACE
ULS23 (2025) [24]	3,029 + 284	83% [23,35,36,52,57]	Multiple organ pathologies

**Abbreviations:** NSCLC, Non-Small Cell Lung Cancer; CRC, Colorectal Cancer; FNH, Focal Nodular Hyperplasia; HCC, Hepatocellular Carcinoma; TACE, Transarterial Chemoembolization; GIST, Gastrointestinal Stromal Tumor; CRLM, Colorectal Liver Metastases; PDAC, Pancreatic Ductal Adenocarcinoma, CCRCC: Clear Cell Renal Cell Carcinoma.

**Table 2:** Dataset centers, organs, and annotation methods.

Dataset name	Centers	Annotated organs	Anomaly label	Annotation
SLIVER (2007) [28]	–	L	–	expert
3D-IRCADb (2010) [29]	1(FR)	L, SV, GB, AO, LES	✓	expert
VISCERAL (2015) [30]	–	L, GB, P, SP, K	–	expert
BTCV (2015) [31]	1(US)	L, SV, GB, ST, P, SP, K, AG, AO, ES	–	expert
Colorectal-Liver-Metastases (2017) [32]	1(US)	L, HV, SV, LES	✓	expert
DenseVNet (2018) [33]	2(US)	UAO	–	expert
LiTS (2018) [35]	7(DE, NL, CA, IL, FR)	L, LES	✓	expert
MSD-CT – Spleen task (2018) [36]	1(US)	SP	✓	AI+expert
Pancreatic Cancer Survival Prediction (2018) [37]	1(US)	P, LES	✓	expert
MSD-CT – Colon task (2018) [36]	1(US)	CO	✓	expert
SegThor (2019) [38]	1(FR)	AO, ES	–	expert
CHAOS (2019) [39]	1(TR)	L	–	expert
CT-ORG (2020) [40]	8(DE, NL, CA, FR, IL, US)	L, K	✓	AI+expert
MSD-CT – Pancreas task (2020) [36]	1(US)	P, LES	✓	expert
MSD-CT – Liver task (2020) [36]	7(DE, NL, CA, IL, FR)	L	✓	expert
MSD-CT – HepaticVessel task (2020) [36]	1(US)	L, HV	✓	AI+expert
Pancreas-CT (2020) [34]	1(US)	P	–	expert
AbdomenCT-1K (2021) [19]	12(DE, NL, FR, IL, US, CA, CN)	L, P, SP, K, CT	✓	AI+expert
WORC – GIST dataset (2021) [41]	1(NL)	LES	✓	expert
WORC – CRLM dataset (2021) [41]	1(NL)	LES	✓	expert
Pediatric (2022) [42]	1(US)	UAO, AG, IN, CO, RE	–	expert
WORD (2022) [43]	1(CN)	UAO, AG, IN, CO, RE	–	expert
AMOS (2022) [44]	2(CN)	UAO, AG, AO	–	AI+expert
KiPA22 (2022)	1(CN)	K, LES	✓	expert
StageII-Colorectal-CT (2022) [46]	1(CN)	LN, LES	✓	expert
HCC-TACE-Seg (2022) [47]	1(US)	L, LES	✓	expert
AutoPET (FDG-PET/CT) (2022) [48]	2(DE)	LES	✓	expert
DAP Atlas (2023) [49]	NA(DE)	SV, AG, IN, CO, RE, AO, MES	✓	AI+expert
Abdominal Trauma Det (2024) [50]	23(More than 10 countries)	UAO, SV, IN, CO, RE, AO, MES	✓	AI+expert
TotalSegmentator (2023) [51]	8(CH)	SV, AG, IN, CO, AO	✓	AI+expert
AbdomenAtlas 1.1 (2024) [21]	112(More than 10 countries)	UAO, HV, SV, AG, IN, CO, RE, AO, CT	✓	AI+expert
FLARE23 (2023) [20]	44(CN, CA, BR, US, DE, FR, IL, PL, UK)	UAO, AG, AO	✓	AI+expert
KiTS (2019) [52]	1(US)	K, LES	✓	expert
CPTAC-PDA-Tumor-Annotations (2023) [53]	NA	LN, LES	✓	AI+expert
CPTAC-CCRCC-Tumor-Annotations (2023) [54]	NA	LN, LES	✓	AI+expert
CARE (2023) [55]	1(CN)	RE, LES	✓	expert
Low-dose (2023) [56]	2(US)	LES	✓	expert
SEG.A. (2023)	NA	AO	–	–
CT Lymph Nodes (2023) [57]	1(US)	LN	–	expert
Adrenal-ACC-Ki67-Seg (2023) [58]	1(US)	LES	✓	AI+expert
AIMI Annotations Initiative (2024) [59]	NA	L, K, LES	✓	AI + expert
CURVAS (2024) [60]	1(DE)	L, P, K	✓	AI+expert
PANORAMA (2025) [61]	13(NL, SE, NO, DE, CA, IL, FR, US)	P, HV, SV, AO, PD, CBD, LES	✓	AI+expert
WAW-TACE (2024) [62]	1(PL)	L, LES	✓	expert
ULS23 (2025) [24]	11 (US, NL, DE, CA,IL, FR)	LES	✓	AI+expert

**Abbreviations:** US, United States; DE, Germany; NL, Netherlands; CA, Canada; IL, Israel; FR, France; TR, Turkey; CN, China; CH, Switzerland; BR, Brazil; PL, Poland; UK, United Kingdom; UAO, Upper Abdominal Organs (L, GB, SP, P, K, DU, ES, ST); L, Liver; HV, Hepatic Vessel; SV, Splenic Vein; GB, Gallbladder; ST, Stomach; P, Pancreas; SP, Spleen; K, Kidney; AG, Adrenal Gland; IN, Intestine; CO, Colon; RE, Rectum; AO, Aorta; CT, Celiac Trunk; MES, Mesentery; LN, Lymph Node; ES, Esophagus; LES, Lesion.

### 3.2 Dataset composition and redundancy

**Fig 1:**
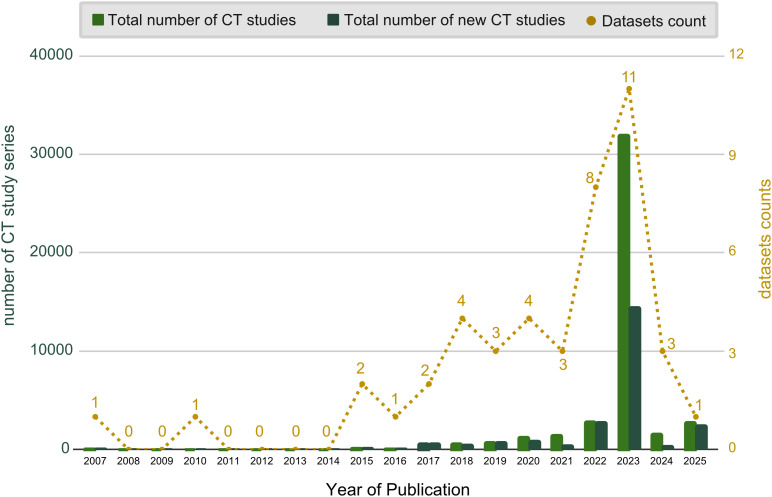
Temporal trends in publicly available abdominal CT dataset publications and case accumulation from 2007 to 2025. Light green bars represent the total number of CT studies released per year, while dark green bars represent the number of new (nonreused) CT studies. The dotted gold line (right axis) indicates the number of datasets published each year. A sharp increase in both dataset releases and total case volume is observed from 2022 onward, peaking at 11 datasets in 2023; however, the widening gap between total and new CT studies in this period reflects increasing case reuse across composite collections.

**Fig 2:**
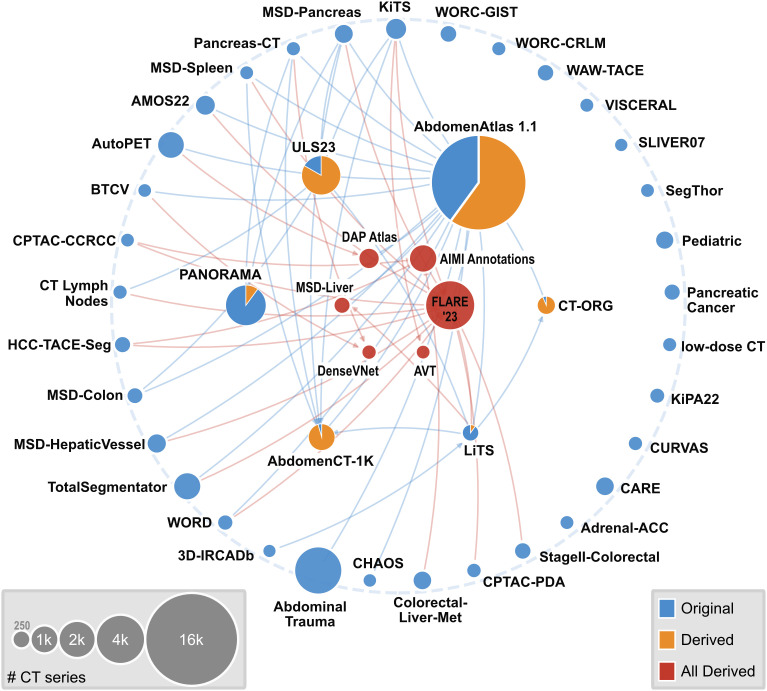
Network of case reuse across publicly available abdominal CT datasets. Each node represents a dataset, with node size proportional to the number of CT series (see legend, lower left). Node colour indicates the proportion of original versus reused cases: blue denotes fully original datasets, orange denotes datasets containing a mixture of original and derived cases, and red denotes datasets composed entirely of cases reused from other collections. Directed edges (arrows) connect source datasets to the derivative collections that incorporate their cases. A small number of foundational datasets, including LiTS, KiTS, Pancreas-CT, and BTCV, function as hub nodes from which cases propagate into multiple downstream composite collections such as AbdomenCT-1K, FLARE’23, and AbdomenAtlas 1.1.

Figure 1 highlights a notable trend in dataset composition—the frequent reuse of the same CT studies across multiple datasets. While this practice can improve resource efficiency, it also reduces data diversity and may hinder the generalizability of AI models to varied clinical scenarios. Among the 47,049 CT studies examined, only 23,746 were original, revealing substantial redundancy. Such overlap raises the risk of data leakage during model training, which can lead to overfitting, where models learn to memorize specific cases rather than develop robust, generalizable representations. The dataset reusage graph is represented in Fig 2 and you can find more details in Table 1.

### 3.3 Geographic distribution of datasets

Publicly available abdominal CT datasets have predominantly been acquired using scanners from major manufacturers such as GE, Siemens, Philips, and Toshiba, with 16- and 64-detector configurations being the most frequently reported systems. Despite contributions from 18 countries, the geographic distribution of datasets is heavily imbalanced, with a clear overrepresentation of high-income regions.

**Fig 3:**
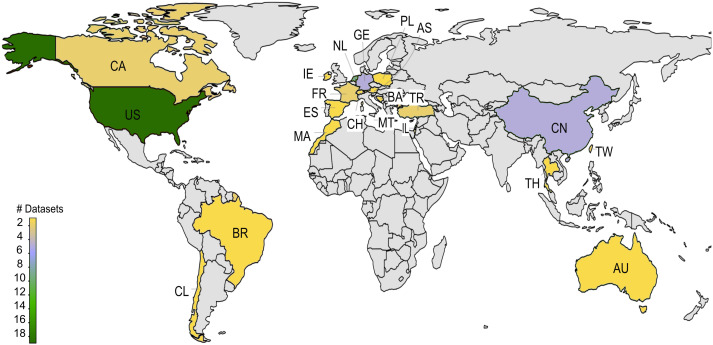
Geographic distribution of contributing countries across publicly available abdominal CT datasets. Base map borders derived from the GSHHG database (https://www.soest.hawaii.edu/pwessel/gshhg), released under the GNU LGPL v3. Countries are shaded according to the number of datasets to which they contribute (colour scale, lower left), with darker green indicating higher representation. North America and Europe collectively account for approximately 75% of dataset contributions. Large regions of the globe, including sub-Saharan Africa, South Asia, and most of the Middle East, have no representation in the current public dataset landscape. Country codes: AS, Austria; AU, Australia; BA, Bosnia and Herzegovina; BR, Brazil; CA, Canada; CH, Switzerland; CL, Chile; CN, China; ES, Spain; FR, France; GE, Germany; IE, Ireland; IL, Israel; MA, Morocco; MT, Malta; NL, Netherlands; PL, Poland; TH, Thailand; TR, Turkey; TW, Taiwan; US, United States.

As shown in Fig 3, ~75% of the datasets originate from the United States, Canada, and European countries, reflecting a strong Western bias. The United States alone accounts for 21% of all datasets, making it the most prolific single contributor, followed by China and France, each contributing 9%. In contrast, datasets from nonWestern regions—including Turkey, Taiwan, Chile, Morocco, Bosnia and Herzegovina, and Brazil—collectively represent only 22% of the total, indicating a substantial underrepresentation of low- and middle-income countries.

Notably, several major global regions—such as most of Africa, South Asia, and the Middle East—are entirely absent from the current dataset landscape. This geographic concentration limits the diversity of imaging sources and raises critical concerns about the generalizability of AI models trained on these datasets. Models developed from such regionally skewed data may underperform when applied in underrepresented healthcare settings, ultimately hindering equitable deployment and clinical utility across global populations.

### 3.4 Organ and pathology distribution

**Fig 4:**
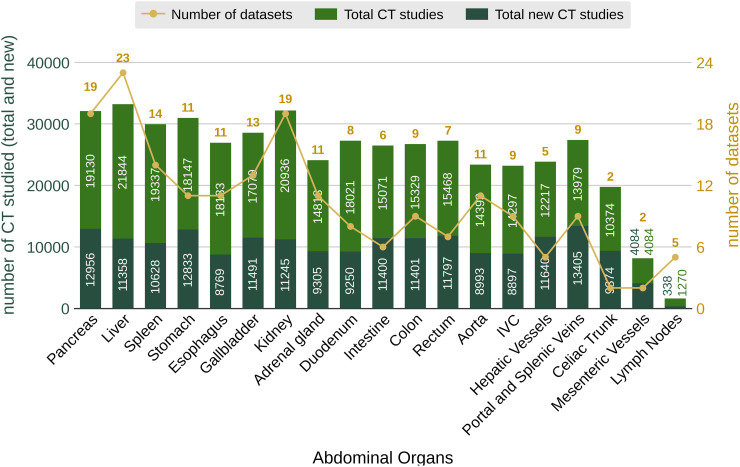
Organ-level representation across publicly available abdominal CT datasets. Light green bars indicate the total number of CT studies containing annotations for each organ (including reused cases), while dark green bars indicate the number of new (unique) CT studies. The gold line (right axis) shows the number of distinct datasets covering each organ. The liver (23 datasets) and kidney (19 datasets) are the most frequently annotated structures. Numeric labels on bars denote exact CT study counts.

Table 3 and Fig 4 summarize the distribution of organ-specific abnormalities across publicly and privately available abdominal CT datasets. The liver and pancreas are the most frequently annotated organs, with rich datasets available for both healthy and diseased states. Liver pathologies span a broad clinical spectrum, including primary hepatic tumors, metastases, trauma-related injuries, and post-treatment imaging—highlighting the liver’s prominence in abdominal imaging research. Likewise, pancreatic datasets frequently include cases of cystic lesions and malignancies, reflecting the organ’s diagnostic complexity and clinical importance.

**Table 3:** Organ segmentation masks and lesion annotations (New CT Counts).

Organ	Mask type	Description/covered anomalies	New CTs
Adrenal gland	Organ Mask	No specific pathology reported	9,305 + 9,575
	Lesion Mask	Adrenocortical carcinoma (pathologically confirmed): Stage I: 4; Stage II: 19; Stage III: 23; Stage IV: 7	65
Aorta	Organ Mask	Abdominal aortic aneurysm and aortic dissection (56 public cases); no specific pathology in remaining cases	8,993 + 9,575
	Lesion Mask	–	–
Celiac trunk	Organ Mask	No specific pathology reported	9,374 + 11,223
	Lesion Mask	–	–
Colon	Organ Mask	BMAI^†^; primary CRC (126 cases); no specific pathology in remaining cases	11,401 + 10,178
	Lesion Mask	CRC Stage II	230
Duodenum	Organ Mask	BMAI^†^; no specific pathology in remaining cases	9,250 + 10,278
	Lesion Mask	–	–
Esophagus	Organ Mask	BMAI^†^; no specific pathology in remaining cases	8,769 + 10,318
	Lesion Mask	–	–
Gallbladder	Organ Mask	Common bile duct annotations in 1,964 public cases; no specific pathology in remaining cases	11,491 + 10,061
	Lesion Mask	–	–
Hepatic Vessels	Organ Mask	Primary and metastatic liver tumours (cholangiocarcinoma, HCC, metastases; 303 cases); CRLM pre- and post-procedure (394 cases); no specific pathology in remaining cases	11,640 + 11,709
	Lesion Mask	–	–
IVC	Organ Mask	No specific pathology reported	8,897 + 9,575
	Lesion Mask	–	–
Intestine	Organ Mask	BMAI^†^ (81 cases); no specific pathology in remaining cases	11,400 + 10,178
	Lesion Mask	–	–
Kidney	Organ Mask	Renal tumours (1,088 cases); cysts (268 cases); traumatic injury (217 cases); no specific pathology in remaining cases	11,245 + 10,530
	Lesion Mask	Cysts	248
		Renal tumours (mixed histology)	499
		Renal cell carcinoma subtypes: clear cell, papillary, chromophobe; angiomyolipoma; eosinophilic adenoma	100
Liver	Organ Mask	*Primary malignancy:* hepatocellular carcinoma (HCC; 448 cases including pre-TACE: 105 and post-TACE: 105). *Metastatic disease:* colorectal liver metastases (CRLM; 197 cases with pre- and post-procedure scans). *Mixed malignant datasets:* primary and metastatic tumours with pre- and post-therapy imaging (194 cases). *Benign:* cysts and focal nodular hyperplasia (FNH). *Trauma:* liver injury (340 + 151 cases). *Mixed pathology:* datasets containing tumours, metastases, cysts, or unspecified benign and malignant lesions.	11,358 + 10,267
	Lesion Mask	Primary and metastatic tumours, pre- and post-therapy	99 + 70
		Hepatocellular carcinoma (HCC)	588
		Colorectal liver metastases (CRLM)	471
		Benign lesions (FNH, cysts) and unspecified liver tumours	92
Lymph Nodes	Organ Mask	LN surrounding CRC (230 cases); noncancerous lymphadenopathy (86 cases); LN diameter >1 cm in PDAC (10 cases) and CRC (12 cases)	338 + 0
	Lesion Mask	–	–
Mesenteric Vessels	Organ Mask	BMAI†	4,084
	Lesion Mask	–	–
Pancreas	Organ Mask	Pancreatic cysts or tumours (460 cases); pancreatic parenchyma and duct annotations (1,964 cases); no specific pathology in remaining cases	12,956 + 10,550
	Lesion Mask	PDAC (positive cases only)	608
		PDAC	90
		Pancreatic cancer (unspecified)	55
Portal and Splenic Veins	Organ Mask	CRLM pre- and post-procedure (394 cases)	11,441 + 9,475
	Lesion Mask	–	–
Rectum	Organ Mask	BMAI^†^; rectal cancer (399 cases); rectal cancer unspecified (37 cases)	11,797 + 10,178
	Lesion Mask	–	–
Spleen	Organ Mask	Splenic injury (372 cases)	10,628 + 10,298
	Lesion Mask	–	–
Stomach	Organ Mask	BMAI^†^	12,833 + 10,298
	Lesion Mask	–	–
*General*	Lesion Mask	Malignant lymphoma, melanoma, NSCLC	501 + 151
		GIST and mimicking tumours (schwannoma, leiomyosarcoma, esophageal/GEJ carcinoma, lymphoma)	223

**Abbreviations:** BMAI, bowel/mesenteric injury with active extravasation (Abdominal Trauma Detection dataset; 3,551 public cases); CRC, colorectal cancer; CRLM, colorectal liver metastases; FNH, focal nodular hyperplasia; GEJ, gastroesophageal junction; GIST, gastrointestinal stromal tumour; HCC, hepatocellular carcinoma; LN, lymph node; NSCLC, nonsmall cell lung cancer; PDAC, pancreatic ductal adenocarcinoma; TACE, transarterial chemoembolisation. New CT counts are reported as public + private where applicable; “–” indicates no dedicated lesion annotations for that organ.

†BMAI cases all originate from the Abdominal Trauma Detection dataset (3,551 public cases).

Abnormalities in the kidneys and spleen are also well-documented, especially in the context of neoplasms, cysts, and trauma. In contrast, although imaging data for the gallbladder and adrenal glands are present in several datasets, the frequency of labeled abnormalities for these organs is markedly lower. This discrepancy may reflect either a lower incidence of clinically significant findings or a lack of detailed annotation in existing resources.

Beyond solid organs, several datasets include colorectal, rectal, and other gastrointestinal lesions, emphasizing the relevance of abdominal CT imaging in oncology applications. Despite this breadth, notable gaps remain. Many common, nonneoplastic conditions—such as inflammatory or vascular diseases—are underrepresented, which may inadvertently bias AI models toward tumor-centric tasks. As a result, these models risk underperforming in more general diagnostic scenarios, limiting their utility in routine clinical practice.

Addressing this imbalance will require broader annotation efforts and the inclusion of diverse pathologies to ensure AI tools are developed with a more comprehensive diagnostic foundation.

### 3.5 Annotation practices and dataset bias

Annotation methodologies vary across datasets, impacting the reliability of AI model training, 60% of datasets rely on manual annotation by radiologists and trained experts and 35% use AI-assisted labeling, where AI-generated annotations are later refined by human experts and the rest are fully AI-annotated, introducing potential concerns regarding labeling accuracy.

While AI-assisted annotation offers efficiency gains, studies suggest that fully AI-generated labels may introduce systematic errors, particularly in complex segmentation tasks [9]. Ensuring annotation consistency across datasets is crucial for reliable AI training.

### 3.6 The bias evaluation revealed substantial disparities in dataset fairness

**Fig 5:**
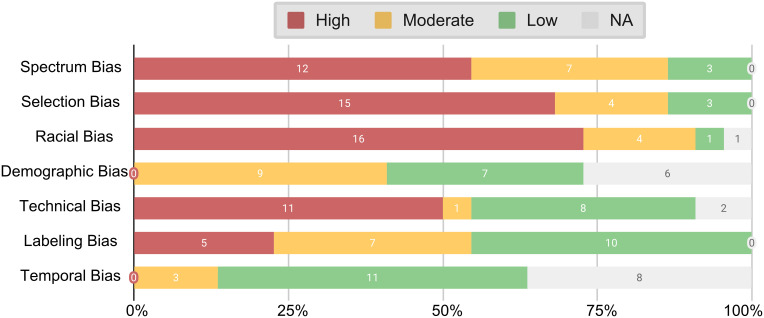
Distribution of bias risk ratings across seven assessment categories for the 22 datasets with 100 or more public cases. Horizontal stacked bars show the number of datasets rated as high risk (red), moderate risk (yellow), low risk (green), or not available (grey) for each bias category. Racial bias (16 high-risk) and selection bias (15 high-risk) were the most prevalent high-risk categories, while labelling bias (10 low-risk) and temporal bias (11 low-risk) were comparatively less affected. Categories rated as not available reflect insufficient documentation in the primary dataset publications. Full per-dataset ratings and operational definitions for each category are provided in Table 4 and S1 Table, respectively.

Table 4 summarizes the bias evaluation, which excluded datasets containing fewer than 100 cases, resulting in a final set of 22 datasets. Figure 5 shows that the most common bias types were domain shift bias (63%) and selection bias (57%), indicating that many datasets may not generalize effectively beyond their original clinical environments. Spectrum bias (52%) and racial bias (52%) were also prevalent, suggesting over-representation of specific pathologies and patient demographics, which may adversely affect model fairness. Labeling bias was least frequent (5.3%), implying that annotation inconsistencies are a comparatively minor concern relative to dataset composition. In total, 47% of datasets (*n* = 9) exhibited three or more high-risk bias indicators, whereas only 10% (*n* = 2) had three or more low-risk ratings. These findings demonstrate significant disparities in fairness and representational validity, underscoring the need for more diverse and balanced datasets to improve AI model generalizability and fairness.

**Table 4:** Risk of bias assessment for datasets with ≥100 cases.

Dataset/Risk of bias	Spectrum	Selection	Racial	Demographic	Technical	Labeling	Temporal	Overall bias
AutoPET (FDG-PET/CT) (2022) [48]	M	H	H	L	H	H	M	Critical
CARE (2023) [55]	H	H	H	M	H	L	L	Critical
Colorectal-Liver-Metastases (2017) [32]	H	H	H	M	H	H	NA	Critical
HCC-TACE-Seg (2022) [47]	H	H	H	M	H	L	L	Critical
KiPA22 (2022) [45]	H	H	H	NA	H	L	L	Critical
KiTS (2019) [52]	H	H	H	M	H	M	M	Critical
MSD-CT – Colon task (2018) [36]	H	H	H	NA	H	H	NA	Critical
MSD-CT – HepaticVessel task (2020) [36]	M	H	H	NA	H	M	NA	Critical
MSD-CT – Pancreas task (2020) [36]	H	H	H	NA	H	H	NA	Critical
Pancreatic Cancer Survival Prediction (2018) [37]	H	H	H	NA	NA	H	NA	Critical
StageII-Colorectal-CT (2022) [46]	H	H	H	M	H	M	NA	Critical
WORD (2022) [43]	H	H	H	M	H	L	NA	Critical
WAW-TACE (2024) [62]	H	H	H	M	M	M	L	High
WORC – GIST dataset (2021) [41]	H	H	H	M	L	M	L	High
AMOS22 (2022) [44]	M	M	H	L	L	L	M	Moderate
LiTS (2018) [35]	M	H	M	NA	L	L	L	Moderate
PANORAMA (2025) [61]	M	M	M	M	L	M	L	Moderate
TotalSegmentator (2023) [51]	L	L	H	L	L	M	L	Moderate
ULS23 [24]	M	M	NA	L	NA	L	L	Moderate
AbdomenAtlas 1.1 (2024) [21]	M	M	M	L	L	L	L	Low
Abdominal Trauma Det (2024) [50]	L	L	L	L	L	L	L	Low
Pediatric (2022) [42]	L	L	M	L	L	L	NA	Low

H/M/L, high/moderate/low risk; NA, information not provided. Overall bias classification based on count of high-risk indicators after applying equivalence formula (see Materials and methods section 2.4).

Table 5 presents the most reliable datasets for AI model training, selected based on dataset size, annotation method, organ coverage, and bias indicators. These datasets contain a large number of annotated volumes, provide comprehensive organ coverage, and demonstrate low bias risk across key evaluation metrics. Most annotations were performed by expert radiologists, ensuring high labeling quality. Overall, these datasets offer well-balanced and diverse samples, increasing the likelihood that AI models trained on them will generalize effectively across varied clinical scenarios.

**Table 5:** Selected Abdominal imaging datasets with balanced coverage and low-to-moderate bias.

Dataset	Cases	Organs	Bias risk	Clinical focus
Abdominal Trauma [50]	3,551	14 Organs	Low	Multi-center traumatic injuries (23 institutions, 10+ countries)
Pediatric [42]	359	12 Organs	Low	One center pediatric (ages 5 days–16 years) cases who required CT scans
AbdomenAtlas 1.1 [21]	9,262	16 Organs	Low	Multi-center normal/pathologic organs (112 institutions, 10+ countries)
PANORAMA [61]	2,238	7 Organs	Moderate	Multi-center pancreatic pathologies (13 institutions, 8 countries)
TotalSegmentator [51]	1,204	5 Organs	Moderate	Multi-center normal/pathologic organs (8 institutions in Chine)
ULS23 [24]	3,029	abdomen organ lesions	Moderate	Multi-center cases with pathologies (11 institutions in multiple countries)
AMOS22 [44]	500	10 Organs	Moderate	Cases with tumors or non-malignant pathologies(2 institutions in China)
LiTS [35]		1 Organ and its lesions	Moderate	Multi-centered liver cancer, pre- and post-therapy (7 institutions in 5 countries)

## 4 Discussion

This review identified three converging findings across 45 publicly available abdominal CT datasets. First, half of the 47,049 catalogued CT studies are cases reused from earlier collections, reducing the original data representation. Second, ~75% of datasets originate from North America and Europe, while sub-Saharan Africa, South Asia, and most of the Middle East contribute none. Third, among the 22 datasets large enough for structured bias assessment, 16 carried high-risk racial bias, with no dataset achieving low risk for selection bias, meaning that no public resource adequately represents the differential diagnoses that clinicians encounter in practice. These findings, taken together, indicate that the data foundations of abdominal imaging AI are narrower, less diverse, and more systematically skewed than their apparent scale implies. The biases documented here are not incidental shortcomings of individual datasets. They are structural properties of the ecosystem that will propagate, by default, into every model trained on these resources.

Our findings confirm that the upstream bias problem proposed in prior commentaries [8,9] is not hypothetical but pervasive and quantifiable. Dataset-level audits in adjacent domains have reached convergent conclusions, with reviews of chest radiograph collections documenting geographic concentration toward North American institutions [15] and audits of dermatological repositories revealing demographic skews that predict downstream diagnostic failures [16]. The present review extends this pattern to abdominal CT and adds a dimension not previously characterised, namely a systematic misalignment between the organs and pathologies the ecosystem provides and those that clinical practice demands. Our bias assessment revealed that selection bias was the only category in which no dataset achieved a low-risk rating. The full spectrum of differential diagnoses encountered in routine practice remains unrepresented across the entire public data landscape. This gap is not random. It reflects a predictable selection pressure in which organs with large volumes and well-defined boundaries yield higher segmentation accuracy, attract disproportionate benchmark attention, and generate more publishable results [63]. Clinically essential structures that are smaller, more irregular, or harder to annotate remain systematically neglected [9].

The structured reuse of cases across the dataset ecosystem warrants particular attention. Nearly half of the 47,049 catalogued studies are recycled from prior collections, and this reuse follows a network topology in which a small number of foundational datasets serve as hub nodes from which cases propagate into multiple derivatives (Fig 2). A researcher who trains on one composite dataset and evaluates on another may therefore test on cases already encountered during training. This phenomenon is consistent with broader trends in machine learning, where benchmark concentration creates a risk of community-level overfitting [63], and in medical imaging specifically introduces an implicit multiple-comparison problem that may inflate reported performance beyond what true generalisation would support [64]. If this concern proves well-founded at scale, mitigation may require new infrastructure such as a public registry of anonymised imaging identifiers to enable systematic deduplication before train-test partitioning.

Beyond redundancy, the geographic concentration of available data poses a clinical, not merely ethical, threat. The absence of datasets from sub-Saharan Africa, South Asia, and much of the Middle East removes entire disease presentations from the training distribution. Hepatocellular carcinoma illustrates this point: in the reviewed datasets, HCC cases derive predominantly from Western populations, where the disease arises in cirrhotic livers secondary to hepatitis C, alcohol-related liver disease, or metabolic steatohepatitis [65], whereas in sub-Saharan Africa and parts of East Asia, HCC frequently develops in noncirrhotic livers driven by chronic hepatitis B and aflatoxin exposure, presenting in younger patients with distinct imaging morphology [66]. A model trained on Western data alone may therefore be systematically blind to what constitutes the predominant disease phenotype in other regions, a mismatch that widens precisely where clinical need is greatest.

The barriers to geographic inclusion are structural. The bottleneck lies not in the absence of imaging in these regions but in the incentives and costs of converting clinical data into public research resources, which requires de-identification systems, ethics frameworks for international sharing, annotation capacity, and long-term hosting [15,67]. A reinforcing feedback loop compounds this asymmetry. Western-dominated benchmarks penalise models developed on local data from underrepresented regions, because domain shift depresses evaluation metrics, reduces competitiveness in peer review, and keeps such data invisible to the broader field [14]. Breaking this cycle will require federated initiatives that enable collaborative model development without raw data export [68], annotation partnerships linking expert centres with local repositories, and evaluation frameworks that reward geographic diversity alongside performance [64].

These geographic and compositional limitations are not independent; they interact through a compounding process that amplifies initial biases at each stage of the dataset lifecycle. This process begins at recruitment, where single-centre convenience sampling produces cohorts that reflect institutional referral patterns rather than the clinical populations that deployed models will serve [9]. The universally elevated risk for selection bias observed in our analysis is a direct consequence, indicating that no public abdominal CT resource adequately captures the differential diagnoses clinicians face in practice, where the diagnostically consequential distinction is not tumour versus normal tissue but tumour versus its clinical mimics. The process continues at acquisition, where single-scanner or narrow-protocol data introduce technical signatures that models absorb as spurious features rather than genuine anatomy [6], and compounds at annotation, where over one-third of reviewed datasets employ AI-assisted pipelines whose reliability depends on models trained on earlier datasets carrying the same biases documented here. This recursion means that initial distortions can amplify across successive dataset generations rather than self-correct [10].

The cumulative effect of narrow recruitment, standardised acquisition, and recursively biased annotation explains why the majority of assessed datasets carry critical or high overall bias. Whether models trained on these resources can be rendered safe for heterogeneous clinical deployment through post-hoc correction alone remains an open question.

This review has several limitations that warrant acknowledgement. The bias assessment framework, while grounded in previous frameworks decisions [18,26,27], is novel and requires further validation. The scoring thresholds involve judgment, and alternative weighting schemes could shift individual datasets between adjacent categories. We therefore provide all per-dataset ratings in the supplementary materials and recommend interpreting the classifications as relative risk rankings rather than absolute determinations. Our assessment captured representation at the dataset level but could not evaluate whether annotation quality varies systematically across demographic subgroups within a given dataset, a form of measurement bias requiring per-case provenance data that public releases rarely include. Domain adaptation offers a partial counterargument to the concerns raised here.

We acknowledge its growing evidence base, yet note three constraints. These methods still require representative target-domain data for calibration, cannot correct for fundamentally different disease spectra through distributional alignment alone, and shift the adaptation burden onto institutions least equipped to bear it. Finally, we recognise that imperfect AI may still yield net clinical benefit in settings entirely lacking expert radiological interpretation. Our critique argues not against deployment in such contexts but for transparency about known limitations and sustained investment in the data infrastructure that would make deployed tools safer and more equitable.

## 5 Conclusion

The findings of this review reframe dataset bias in medical imaging as an upstream structural problem rather than a downstream model behaviour. Redundancy, geographic exclusion, and demographic narrowness are not properties of individual datasets that can be patched in isolation. They are emergent features of an ecosystem shaped by convenience sampling, institutional concentration, and misaligned incentives, and they will persist until the field treats representative data collection with the same rigour it applies to model development and validation. Prospective collection, transparent documentation, and shared infrastructure for deduplication and cross-domain evaluation offer a path forward, but only if these efforts deliberately include the institutions and patient populations that current resources leave out. Until then, the promise of AI in medical imaging may reach furthest where it is needed least, and fall shortest where it is needed most.

## Supporting information

S1 Table Operational definitions and evaluation criteria for the bias assessment framework. This table provides the detailed definitions of the seven bias categories (spectrum, selection, racial, demographic, technical, labelling, and temporal), together with the exact criteria used to assign low, moderate, and high risk ratings.
(PDF)

S2 Table Detailed per-dataset bias assessment with reviewer justifications (*n* = 22 datasets with ≥100 public cases). Comprehensive evaluation including risk rating and written justification for each bias category and each dataset. Presented in two parts for readability (landscape format).
(PDF)

S3 TableInter-rater reliability statistics for the bias assessment.Three-rater agreement metrics (Krippendorff’s α and quadratic-weighted Cohen’s κ), pairwise rater agreement, and annotator-to-adjudicated-ground-truth agreement across all seven bias categories (sub-tables S3a, S3b, S3c).(PDF)

S1 Fig Dataset identification and selection flow diagram. PRISMA-style flow chart showing the systematic search, screening, eligibility assessment, and final inclusion of abdominal CT datasets.
(PDF)
