# Supplementary material for: State of abdominal CT datasets: A critical review of bias, clinical relevance, and real-world applicability
Source: PLOS Digit Health. 2026 Aug 10;5(8):e0001567. doi: 10.1371/journal.pdig.0001567 (PMC13456201; doi:10.1371/journal.pdig.0001567)
Supplement: Supplementary file 1 [file pdig.0001567.s001.pdf]

S1 Table

Bias Category Definitions and Assessment Criteria

The bias assessment framework was developed by adapting instruments from PROBAST-AI, the quality criteria for medical imaging AI datasets proposed by Kocak et al. [1], and the TRIPOD+AI reporting guidelines. Seven bias categories were defined, each addressing a distinct dimension of dataset quality. These categories collectively inform three higher-order threats to clinical applicability: case bias (spectrum and selection bias), population bias (racial and demographic bias), and domain shift bias (technical, temporal, and selection bias). Table 1 provides the operational definitions and three-tier rating criteria for each category.

Table 1: Operational Definitions and Risk Level Criteria for Bias Assessment Categories

Bias Category	Definition	Low Risk	Moderate Risk	High Risk
**Spectrum Bias** (Case Representation)	Evaluates the severity range and clinical variation within the positive (pathological) class relative to the spectrum encountered in routine practice.	Covers most clinically important variations of the target condition, including multiple severity levels, stages, or presentations (e.g., for pancreatic cancer: resectable, locally advanced, and metastatic disease).	Missing one or two clinically important presentations or severity levels within the positive class.	Heavily skewed representation with many clinically significant severities and presentations absent (e.g., only a single disease stage or post-operative cases only).
**Selection Bias** (Non-Case Representation)	Evaluates the spectrum of non-case (negative class) variation and the coverage of clinically relevant differential diagnoses.	Includes most relevant differentials in the non-case group (e.g., for pancreatic cancer: pancreatic cysts, other malignancies, normal pancreata, and important incidental findings).	Missing one or two clinically important differential diagnoses in the non-case group.	Many differential diagnoses absent, or only a single disease entity in the non-case group, or only normal cases serving as the non-case comparator.
**Racial Bias**	Evaluates the coverage of racial and ethnic groups relative to the intended scope of use (global or multi-country deployment).	Most major racial and ethnic groups represented. Multi-country dataset spanning three or more continents, or explicit documentation of ethnic diversity.	Missing one important racial or ethnic group within the global demographic landscape.	Many racial groups absent, or only a single racial group represented, or single-institution dataset from a racially homogeneous catchment area with no documentation of ethnic composition.
**Demographic Bias**	Evaluates the balance of sex and age distributions within the dataset relative to the clinical population.	Approximately balanced sex distribution and all clinically relevant age groups represented.	Mild imbalance in sex distribution or partial under-representation of one age group.	Heavily skewed sex distribution or an entire clinically relevant age group absent (gender ratio of more than 7-to-3 ratio).

Continued on next page

Table 1 – *Continued from previous page*

Bias Category	Definition	Low Risk	Moderate Risk	High Risk
**Technical Bias** (Protocol Bias)	Evaluates the diversity of imaging hardware, acquisition protocols, contrast phases, and reconstruction parameters relative to routine clinical practice.	Multiple scanner manufacturers and models documented. Explicit variation in contrast phases, slice thickness, and acquisition parameters reflecting real-world heterogeneity.	Limited variation in scanner models or acquisition protocols, but more than one configuration represented.	Single scanner model or exclusively high-specification research equipment not representative of routine clinical settings. No variation in contrast phases, slice thickness, or acquisition parameters.
**Labelling Bias** (Annotation Bias)	Evaluates the rigour and consistency of the annotation pipeline, including the qualifications of annotators, the number of reviewers, and the quality assurance procedures applied.	At least two human reviewers involved and all cases underwent expert validation, either through fully manual annotation or through a structured human-in-the-loop workflow with complete expert review.	Single annotator who is not a domain expert, or AI-assisted pipeline with partial (but not complete) expert review of cases.	Minimal expert supervision, annotators lacking medical training, or fully automated pipeline with no reported accuracy against expert-validated reference data.
**Temporal Bias**	Evaluates the time span and period of data collection. Clinical routines, case representation, and imaging quality can change substantially over short periods.	Data collection spanning more than five years across all contributing sources, capturing natural temporal variation in clinical practice and imaging technology.	Data collection spanning an intermediate period, or collection window partially overlapping with years of atypical clinical practice alongside other years.	All data collected within a single year, or collection period falling entirely within the COVID-19 pandemic years (2019–2021), during which altered clinical workflows and diagnostic thresholds may have affected both case representation and imaging quality.

Overall Bias Classification and Equivalence Formula

Overall bias classifications were derived from per-category ratings using a weighted composite scoring system. The design of this system was informed by three established principles in evidence quality assessment. First, the multi-domain assessment structure of ROBINS-I [2], in which bias is evaluated independently across multiple distinct domains rather than as a single global judgment, provided the structural basis for the seven-category framework. Second, the cumulative downgrading logic of the GRADE framework, in which moderate concerns distributed across multiple independent domains progressively reduce confidence even in the absence of any single severe limitation [3], was operationalised through a coefficient of $\frac{1}{3}$ for moderate-risk ratings, such that three moderate concerns contribute equivalently to one high-risk indicator. Third, the principle from QUADAS-2 that undocumented domains cannot be assumed low risk [4] was applied by treating all categories recorded as "not available" (NA) as Moderate risk for the purposes of composite score computation. This conservative but not punitive assignment reflects the fact that incomplete reporting may conceal problematic characteristics, while avoiding the assumption that undocumented domains are necessarily high risk. The specific coefficients and proportional classification thresholds represent a novel quantitative operationalisation of these principles for dataset-level bias assessment.

For each dataset, a continuous bias score was computed as:

$$\text{Bias Score} = \sum(\text{High-risk ratings}) \times 1.0 \; + \; \sum(\text{Moderate-risk ratings} + \text{NA categories}) \times \frac{1}{3}$$

Classification thresholds were anchored proportionally to the structure of the seven-category assessment framework. Each threshold corresponds to the fraction of the total framework that is effectively compromised, ensuring

that the classification scales with the instrument rather than depending on arbitrary cutpoints. The thresholds were applied as follows:

Overall Classification	Score Range	Proportional Interpretation
Critical Bias	≥ 3.0	Approximately 3/7 of assessed domains at high-risk equivalent, indicating that nearly half the framework is compromised
High Bias	≥ 2.0 and < 3.0	Approximately 2/7 of assessed domains at high-risk equivalent, indicating substantial limitations likely to affect generalisability
Moderate Bias	≥ 1.0 and < 2.0	Approximately 1/7 of assessed domains at high-risk equivalent, indicating some limitations that may affect specific use cases
Low Bias	< 1.0	Less than one domain at high-risk equivalent, indicating minor limitations unlikely to affect most applications

Worked Example

Consider a dataset receiving 1 high-risk rating, 4 moderate-risk ratings, and 2 NA categories. Because NA is treated as Moderate, the effective distribution is 1 high-risk and 6 moderate-risk ratings. The bias score is calculated as:

$$(1 \times 1.0) + (6 \times \frac{1}{3}) = 1.0 + 2.0 = 3.0$$

Because 3.0 meets the threshold of ≥ 3.0, this dataset is classified as **Critical Bias**.

Higher-Order Bias Mapping

The seven rated categories can be conceptually grouped into three higher-order threats to clinical applicability. These groupings were not used in the composite score computation, which treats all seven categories independently. They are provided here as a conceptual framework to assist future users in interpreting per-category ratings in relation to specific deployment concerns.

Higher-Order Threat	Contributing Categories
Case Bias (real-world case/non-case discrimination)	Spectrum Bias, Selection Bias, Labelling Bias
Population Bias (subgroup performance degradation)	Racial Bias, Demographic Bias
Domain Shift Bias (cross-centre/temporal generalisation)	Technical Bias, Temporal Bias

References

[1] Koçak B, Ponsiglione A, Stanzione A, Bluethgen C, Santinha J, Ugga L, et al. Bias in Artificial Intelligence for Medical Imaging: Fundamentals, Detection, Avoidance, Mitigation, Challenges, Ethics, Prospects. Diagnostic and Interventional Radiology. 2024.

[2] Sterne JAC, Hernán MA, Reeves BC, Savović J, Berkman ND, Viswanathan M, et al. ROBINS-I: a tool for assessing risk of bias in non-randomised studies of interventions. BMJ. 2016;355:i4919.

[3] Schünemann HJ, Cuello C, Akl EA, Mustafa RA, Meerpohl JJ, Thayer K, et al. GRADE guidelines: 18. How ROBINS-I and other tools to assess risk of bias in nonrandomized studies should be used to rate the certainty of a body of evidence. Journal of Clinical Epidemiology. 2019;111:105-114.

[4] Whiting PF, Rutjes AWS, Westwood ME, Mallett S, Deeks JJ, Reitsma JB, et al. QUADAS-2: A Revised Tool for the Quality Assessment of Diagnostic Accuracy Studies. Annals of Internal Medicine. 2011;155(8):529-36.
