# Supplementary material for: State of abdominal CT datasets: A critical review of bias, clinical relevance, and real-world applicability
Source: PLOS Digit Health. 2026 Aug 10;5(8):e0001567. doi: 10.1371/journal.pdig.0001567 (PMC13456201; doi:10.1371/journal.pdig.0001567)
Supplement: Supplementary file 2 [file pdig.0001567.s002.pdf]

S2 Table

Detailed Bias Assessment for Datasets with ≥100 Cases (n=22)

Table 1: Comprehensive Bias Evaluation with Reviewer Justifications - Part 1

Dataset	Spectrum	Spectrum Description	Selection	Selection Description	Racial	Racial Description
AbdomenAtlas 1.1	Moderate	Final: No direct report on spectrum of tumors, size, malignancy, or stage, although they reported inclusion of various abnormal conditions.	Moderate	There are 40% negative cases (8K CT volumes), but considering inclusion of more than 20 organs, most negatives appear to be normal cases rather than other abnormalities.	Moderate	No specific report on race; most datasets originate from the USA, Canada, and some European countries.
Abdominal Trauma Det	Low	Trauma patients with possible other pathologies were included.	Low	Negative cases across centers and other pathologies were included.	Low	23 sites in 14 countries across six continents.
AMOS22	Moderate	Although only cancer patients were included, the dataset covers full CT scans of 15 organs with tumors in different organs.	Moderate	Two hospitals with similar imaging protocols but different specialty focus.	High	Single center in China.
AutoPET (FDG-PET/CT)	Moderate	Limited to melanoma, lymphoma, or lung cancer patients who were candidates for FDG-PET acquisition.	High	Only two centers and even the negative cases were suspected of cancer.	High	Two centers within one region in Germany.
CARE	High	Only patients with rectal cancer were included.	High	No negative non-cancer controls.	High	One center in China.
Colorectal-Liver-Metastases	High	Operable colorectal liver metastases.	High	Surgical cases only with no control variations.	High	Single center in the US.
HCC-TACE-Seg	High	Strict inclusion and exclusion criteria as defined on the TCIA webpage.	High	No healthy controls or non-HCC liver disease.	High	Single center in the US.
KiPA22	High	Moderate within renal pathologies but high within abdominal pathologies.	High	Every patient has exactly one diseased kidney with no controls.	High	Single center in China.
KiTS	High	Moderate diversity within renal pathology but limited within abdominal spectrum.	High	Only post-operative CT of kidney tumor patients.	High	Single center in the US; 87.7% Caucasian.
LiTS	Moderate	Mix of hepatocellular carcinoma and metastatic liver disease.	High	Almost all scans contain tumor or suspected tumor.	Moderate	Seven centers across America and Europe.
MSD-CT Colon	High	Only patients with resectable primary cancer were included.	High	No healthy controls or non-disease cases.	High	Single private cancer center.
MSD-CT HepaticVessel	Moderate	Limited description of pathology diversity.	High	Absence of healthy controls.	High	Single private cancer center.
MSD-CT Pancreas	High	High bias within abdominal datasets, moderate within pancreas datasets.	High	No healthy controls included.	High	Likely single center study.
Pancreatic Cancer Survival Prediction	High	Resectable pancreatic cancer patients within one center with available data.	High	Strict inclusion criteria; no healthy controls.	High	Single US cancer center.
PANORAMA	Moderate	Only PDAC-like and healthy cases included.	Moderate	Includes 1103 PDAC and 2337 non-PDAC cases but excludes other differential diagnoses.	Moderate	Multi-center dataset across Europe and the US.
Pediatric	Low	Dataset specifically for pediatric patients (age 5 days to 16 years).	Low	Hospital-based population requiring CT examinations.	Moderate	Two centers in the US.
StageII-Colorectal-CT	High	Single-stage disease cohort (Stage II only).	High	Strict inclusion criteria defined on TCIA.	High	Single center in China.

Dataset	Spectrum	Spectrum Description	Selection	Selection Description	Racial	Racial Description
TotalSegmentator	Low	Includes many pathologies and healthy cases.	Low	Excluded ambiguous cases but total exclusions are small (5%).	High	Single center in Switzerland.
ULS23	Moderate	Many lesions covered but several remain uncovered.	Moderate	Normal anatomy without lesions is underrepresented.	NA	Not reported.
WAW-TACE	High	Only HCC patients eligible for TACE were included.	High	No healthy controls or incidental tumor cases.	High	Single center in Poland.
WORC GIST	High	Mostly GIST-like lesions.	High	No healthy or incidental tumor cases.	High	Single center in Netherlands.
WORD	High	Only radiotherapy candidates with rectal, prostate, or cervical cancer were included.	High	No healthy controls or incidental tumor cases.	High	Single center in China.

Table 2: Comprehensive Bias Evaluation with Reviewer Justifications - Part 2

Dataset	Demographic	Demographic Description	Technical Bias	Technical Description	Labeling Bias	Labeling Description
AbdomenAtlas 1.1	Low	Because of multi-center origin demographic diversity is possible.	Low	Different machines and techniques.	Low	AI-in-the-loop labeling refined by junior radiologists and audited by senior radiologists.
Abdominal Trauma Det	Low	Various sites and demographic characteristics.	Low	Different machines and technologies.	Low	Pool of 43 expert radiologists using majority voting with adjudicator.
AMOS22	Low	Balanced gender distribution.	Low	Eight scanners with different devices.	Low	Machine-generated coarse labels refined by junior radiologists and reviewed by experts.
AutoPET (FDG-PET/CT)	Low	Age 11–95 with almost equal gender prevalence.	High	High-end devices from two centers only.	High	One annotator per center.
CARE	Moderate	65% male with single ethnic/geographic population.	High	Enhanced CT with narrow slice thickness options.	Low	Initial segmentation by surgeons then revised by oncologists.
Colorectal-Liver-Metastases	Moderate	Age 30–88 with 59% male dominance.	High	Single device with fixed settings.	High	Semi-automated segmentation followed by radiologist correction.
HCC-TACE-Seg	Moderate	Male predominance and older patient population.	High	Single device with different detectors and slice thickness.	Low	Three radiology residents supervised by senior radiologist.
KiPA22	NA	Not reported.	High	Single device with fixed settings.	Low	Semi-automatic labeling refined with voting by three experts.
KiTS	Moderate	Mean age 60 years; 40% female.	High	Single vendor imaging.	Moderate	Only disagreement cases between trainee labeling and nnUNet reviewed by experts.
LiTS	NA	Not reported.	Low	Multi-vendor variability.	Low	Segmented by one radiologist and verified by three others.
MSD-CT Colon	NA	Not reported.	High	Single vendor imaging.	High	Single annotator without quality control.
MSD-CT HepaticVessel	NA	Not reported.	High	GE Lightspeed 16 and VCT scanners.	Moderate	Semi-automatic annotation reviewed by radiologist.
MSD-CT Pancreas	NA	Not reported.	High	Two devices from one manufacturer with fixed protocols.	High	Single expert annotator without inter-rater reliability.
Pancreatic Cancer Survival Prediction	NA	Not reported.	NA	Not reported.	High	Single expert annotator.
PANORAMA	Moderate	Age 57–72 across Europe and US populations.	Low	Six different devices with different settings.	Moderate	Manual annotation by radiologists with expert supervision.
Pediatric	Low	Within pediatric age range.	Low	Three devices with different acquisition techniques.	Low	Four experts supervised by a senior radiologist.
StageII-Colorectal-CT	Moderate	Both genders included with mean age 59.7 $\pm$ 11.9.	High	Two devices with similar settings.	Moderate	Resident annotations verified by senior radiologist.
TotalSegmentator	Low	Proper age and sex coverage.	Low	Multiple devices with different settings.	Moderate	Semi-automated labeling refined under clinician supervision.
ULS23	Low	Multiple sites with balanced gender ratio.	NA	Device variability across combined datasets.	Low	Six trained annotators supervised by expert radiologist.
WAW-TACE	Moderate	Age 28–86 with male predominance.	Moderate	Four scanners with different settings.	Moderate	Organs segmented by TotalSegmentator and tumors manually by radiologists.
WORC GIST	Moderate	53% male dominance.	Low	Four devices with varying settings.	Moderate	Manual segmentation by clinicians under radiologist supervision.
WORD	Moderate	Male skew.	High	Single high-resolution device.	Low	Radiologist annotations checked by experts.
