# Supplementary material for: State of abdominal CT datasets: A critical review of bias, clinical relevance, and real-world applicability
Source: PLOS Digit Health. 2026 Aug 10;5(8):e0001567. doi: 10.1371/journal.pdig.0001567 (PMC13456201; doi:10.1371/journal.pdig.0001567)
Supplement: Supplementary file 3 [file pdig.0001567.s003.pdf]

S3 Table

Inter-Rater Reliability of Bias Assessment

Three raters independently assessed 22 datasets across seven bias categories using a four-level ordinal scale (Low, Moderate, High, Critical). Categories with insufficient documentation were recorded as Not Available (NA) and treated as Moderate for metric computation, reflecting a neutral uninformed position [1].

Disagreements were resolved by a senior reviewer; the adjudicated labels constitute the ground truth reported in Table 4.

Metrics. Krippendorff's α (ordinal distance function) is the primary metric: it accounts for the ordered scale and handles missing values natively [2]. Mean pairwise quadratic-weighted Cohen's κ is reported as a complement [3].

Table S3a. Three-rater inter-rater reliability ($n = 22$ datasets).

Bias Category	Krippendorff's α	Mean weighted κ
Spectrum	0.809	0.823
Selection	0.820	0.755
Racial	0.683	0.705
Technical	0.692	0.691
Labeling	0.758	0.728
Demographic	0.302*	0.371*
Temporal	0.199*	0.190*
**Overall**	**0.727**	**0.721**

* Low reliability driven by high NA rates (Demographic: 32% of ratings; Temporal: 55% of ratings) caused by absent metadata in dataset documentation, not by substantive rater disagreement.

Table S3b. Pairwise quadratic-weighted Cohen's κ between individual rater pairs.

Bias Category	R1–R2	R1–R3	R2–R3	Mean
Spectrum	0.737	0.843	0.887	0.823
Selection	0.739	0.645	0.881	0.755
Racial	0.635	0.626	0.856	0.705
Technical	0.714	0.645	0.715	0.691
Labeling	0.593	0.593	1.000	0.728
Demographic	0.306	0.162	0.645	0.371
Temporal	0.377	0.154	0.039	0.190
**Overall**	0.681	0.649	0.835	**0.721**

Table S3c. Agreement between each annotator and the adjudicated ground truth (quadratic-weighted κ).

Bias Category	R1	R2	R3	Mean
Spectrum	0.835	0.879	0.918	0.877
Selection	0.645	0.881	0.913	0.813
Racial	0.662	0.820	0.891	0.791
Technical	0.875	0.815	0.797	0.829
Labeling	0.726	0.894	0.894	0.838
Demographic	0.255	0.713	0.574	0.514
Temporal	0.182	0.545	0.455	0.394
**Overall**	0.712	0.866	0.860	**0.812**

Overall annotator-to-resolved agreement was strong (mean $\kappa = 0.812$), confirming the validity of the adjudicated labels.

References

[1] Whiting PF, Rutjes AWS, Westwood ME, Mallett S, Deeks JJ, Reitsma JB, et al. QUADAS-2: A Revised Tool for the Quality Assessment of Diagnostic Accuracy Studies. Annals of Internal Medicine. 2011;155(8):529-36.

[2] Krippendorff K. Content Analysis: An Introduction to Its Methodology. 2nd ed. Thousand Oaks, CA: Sage Publications; 2004.

[3] Landis JR, Koch GG. The Measurement of Observer Agreement for Categorical Data. Biometrics. 1977;33(1):159-174.
