# Supplementary material for: State of abdominal CT datasets: A critical review of bias, clinical relevance, and real-world applicability
Source: PLOS Digit Health. 2026 Aug 10;5(8):e0001567. doi: 10.1371/journal.pdig.0001567 (PMC13456201; doi:10.1371/journal.pdig.0001567)
Supplement: Supplementary file 4 [file pdig.0001567.s004.pdf]

S1 Fig

Dataset Selection Flow Diagram

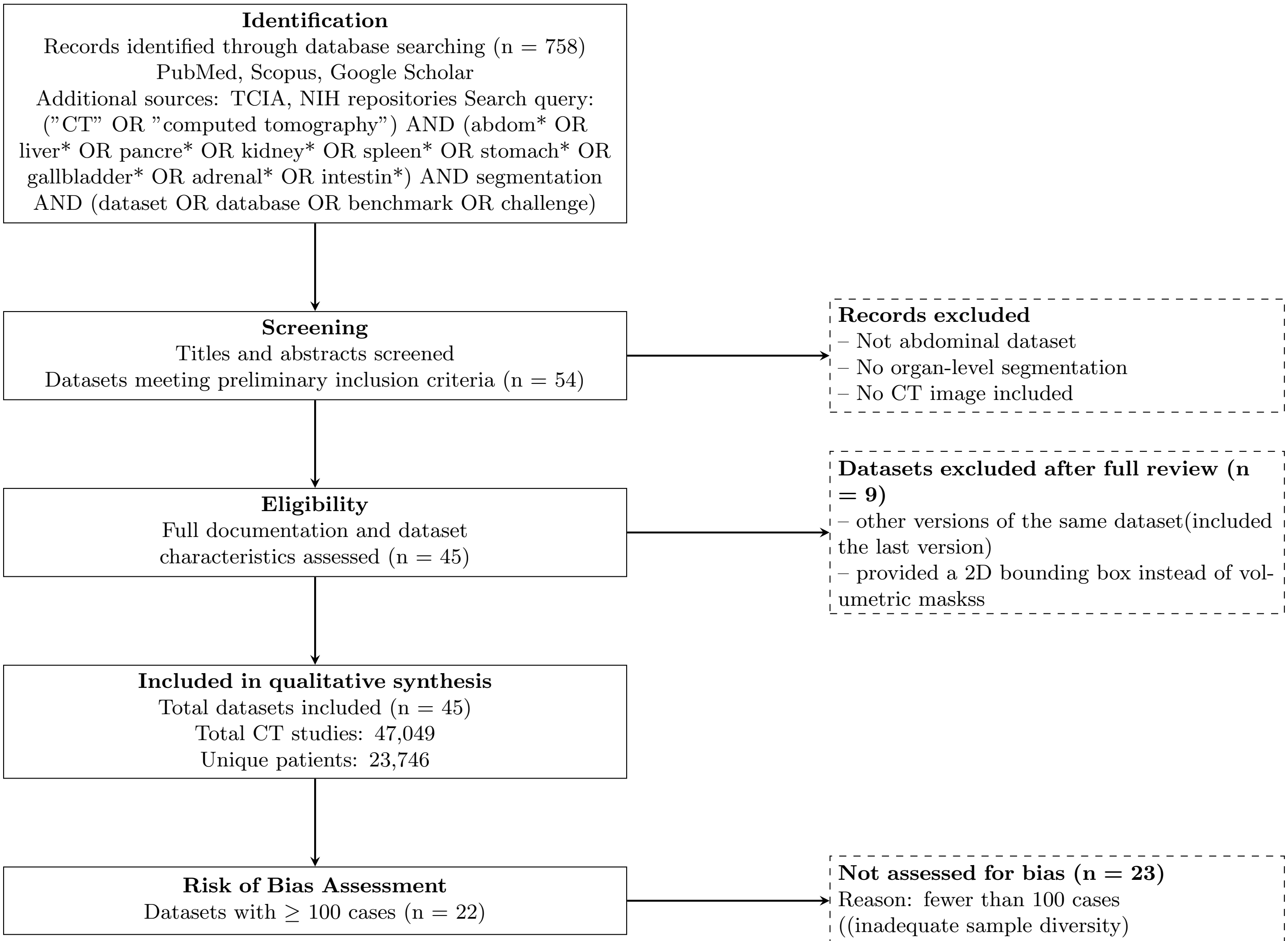

Note: The search was conducted over a two-month period with iterative refinement through co-author consensus. Exclusion decisions were based on predefined criteria described in Section 2.2.
